# Advancing radiation oncology care in Ukraine during the war: impact of international observerships on professional development and clinical practice

**DOI:** 10.3389/fonc.2026.1752691

**Published:** 2026-03-25

**Authors:** Yuliia Lozko, Andriy Beznosenko, Natalka Suchowerska, Viktor Iakovenko, Ruslan Zelinskyi, Zoia Shepil, Serhii Brovchuk, Roman Kowalchuk, Eric Ford, Ben Li, Jonathan Strauss, Indra J. Das, Jan Seuntjens, Rebecca K. S. Wong, Dylan Breiktreutz, Stephen Avery, Akila N. Viswanathan, Trent Aland, Ross Koscharsky, Anthony Walsh, Allen Chen, Daniel T. Chang, Quynh-Thu Le, Nataliya Kovalchuk

**Affiliations:** 1 Surgery Department, National Cancer Institute, Kyiv, Ukraine; 2 Oncology Department, University of Sydney, Sydney, ON, Australia; 3 Oncology Department, University of Texas (UT) Southwestern Medical Center, Dallas, TX, United States; 4 Oncology Department, Spizhenko Clinic, Kyiv, Ukraine; 5 Oncology Department, Shalimov Institute, Kyiv, Ukraine; 6 Oncology Department, Mayo Clinic, Rochester, MN, United States; 7 Oncology Department, University of Washington, Seattle, WA, United States; 8 Oncology Department, Northwestern University Feinberg School of Medicine, Chicago, IL, United States; 9 Oncology Department, The Princess Margaret Cancer Center, ON, Toronto, Canada; 10 Oncology Department, Sunnybrook, Hospital, North York, ON, Canada; 11 Oncology Department, University of Pennsylvania, Philadelphia, PA, United States; 12 Oncology Department, Johns Hopkins University School of Medicine, Baltimore, MD, United States; 13 Oncology Department, ICON Group, Brisbane, QLD, Australia; 14 Oncology Department, University of California, Irvine, Irvine, CA, United States; 15 Oncology Department, Stanford University, Stanford, CA, United States

**Keywords:** fellowship, medical physics, observership, radiation oncology, war, Ukraine

## Abstract

**Objective:**

This study evaluates the impact of international observerships organized by Help Ukraine Group (HUG) on professional development, knowledge transfer, and clinical practice improvement in radiation oncology in Ukraine.

**Methods:**

A total of 50 observerships were facilitated for Ukrainian medical professionals at 14 institutions across the USA, Canada, Europe, and Australia from 2022 to 2024, including 31 radiation oncologists and medical physicists. A survey assessed their impact on oncology care, focusing on knowledge gained, lessons learned, and challenges in implementing new techniques. The primary outcome was the success score, defined as a composite score of implementing new procedures, initiating improvement projects, and knowledge dissemination efforts. Descriptive and comparative analyses examined satisfaction and success outcomes.

**Results:**

A total of 43 respondents participated in the survey (response rate 86.0%). Twenty-eight of the responders were radiation oncology professionals (67.8% radiation oncologists, 32.1% medical physicists). The median observership duration for radiation oncology professionals was four weeks, with 96.4% also attending a professional conference. Overall satisfaction was high (mean 9.5 out of 10, median 10.0, IQR 9.0-10.0) while the success score was substantial (mean 6.1 out of 10, median 7.0, IQR 5.0-8.0). Time since certification influenced success, with ≥10 years’ experience associated with higher success scores (7.4 vs. 4.7, p=0.01). Importantly, 100% of respondents learned new procedures, 89.3% reported shifts in their clinical perspective, and 60.7% successfully implemented new techniques. Subsequent knowledge dissemination in Ukraine was substantial: 82.1% provided informal training, 60.7% delivered institutional or national presentations, 39.3% incorporated materials into lectures, and 10.7% engaged in all activities. Continued mentorship with host institutions was reported by 82.1%.

**Conclusions:**

Amid the war, international observerships advanced clinical practice, education, and procedures in Ukraine, demonstrating the resilience of its medical community and providing a model that other LMICs/UMICs can follow.

## Introduction

The full-scale Russian invasion of Ukraine on February 24, 2022, triggered the largest humanitarian crisis in Europe since World War II. Millions were displaced, and the healthcare infrastructure sustained devastating damage, with more than 2,000 targeted attacks reported (1). Even prior to the invasion, Ukraine’s radiation oncology system faced substantial challenges, including one of the lowest densities of megavoltage radiotherapy (RT) machines in Europe—just 1.7 units per million people (2). The occupation of Crimea and Donbas since 2014 had already resulted in the loss of 10 RT centers and 18 (17%) external beam radiotherapy (EBRT) machines (3). Compounding this was the country’s heavy reliance on aging Co-60 machines (4), most of which were manufactured in Russia with sources irreplaceable under current circumstances.

Additional systemic gaps included 16 high-dose-rate (HDR) afterloaders exceeding 25 years of use and urgently requiring replacement, 13 of 42 unoccupied RT centers (31%) operating without access to a CT scanner, and just three PET/CT scanners available nationwide (5). These infrastructure limitations were dramatically worsened by the war, which temporarily halted radiotherapy services in early 2022, disrupted routine source exchanges, and impeded equipment operation during widespread power outages caused by missile attacks (6–8). As a result, RT patient volumes declined by 5,500 cases in 2022—a drop of 11% compared to 2021 (8). Human resource redistribution also intensified, with 68% of regions reporting workforce redistributions exceeding 25% (8).

Despite these unprecedented challenges, Ukraine’s radiation oncology community demonstrated exceptional resilience. Through resilience and dedication to maintaining cancer care during crisis, treatment volumes in 2023 rebounded to pre-war levels, underscoring the strength and adaptability of Ukraine’s RT professionals (8). In parallel, Ukrainian radiotherapy is also modernizing at a rapid pace. Since the start of the full-scale invasion, 24 new linear accelerators have been installed, and more have been purchased for future deployment. This rapid modernization underscores an urgent need for workforce training, particularly as the national transition from cobalt-based therapy to intensity-modulated radiation therapy (IMRT) accelerates. Significant gaps in knowledge and confidence remain across many areas of modern RT practice, emphasizing the importance of targeted professional development.

Recognizing the urgent need for support, Help Ukraine Group (HUG), in partnership with institutions and other non-profit organizations, including Union for International Cancer Control (UICC), Global Medical Knowledge Alliance (GMKA), Peace and Development Foundation, Stanford Global Scholars at Risk, Nova Ukraine, Future Ukraine Ltd., launched targeted observerships for Ukrainian radiation oncology professionals. These programs provided exposure to modern radiotherapy techniques, multidisciplinary care models, and clinical decision-making strategies at leading cancer centers in the USA, Canada, Europe, and Australia.

International observerships have historically served as powerful tools for knowledge exchange, particularly for clinicians from low- and middle-income countries (LMICs) and upper-middle-income countries (UMICs) (9–12). They offer immersive learning environments where professionals gain direct insight into state-of-the-art technologies, quality assurance protocols, and patient-centered care approaches. They are also vital in establishing ongoing collaborations and partnerships. However, the role of such observerships in strengthening oncology services during wartime remains poorly studied.

This study evaluates the impact of international observerships organized by HUG on radiation oncology professionals from Ukraine. Specifically, it investigates how these programs supported clinical skill development, promoted knowledge dissemination, and facilitated institutional improvements and collaborations in cancer care delivery during wartime. It further explores the barriers faced in implementing new techniques upon return and highlights opportunities for optimizing future training initiatives.

## Methods

Between 2022 and 2024, a total of 50 international observerships were organized by HUG for Ukrainian radiation oncology professionals in partnership with 14 academic and clinical institutions across the United States, Canada, Europe, and Australia. The observers included 31 radiation oncologists and medical physicists who were selected through a structured application and interview process. The WEIGHT consensus guidelines (13) were followed to structure the observerships, emphasizing host-defined needs, appropriate supervision and scope of practice, pre-departure preparation, financial support to reduce host burden, clear learning objectives, structured evaluation, and bidirectional benefit. Observer selection criteria prioritized clinical need, potential for knowledge transfer, and prior experience in education or training, in line with a train-the-trainer model (14). Participants were encouraged from the outset to share their learnings with colleagues upon return to Ukraine, both informally within their departments and formally through presentations, lectures, and training sessions. Many selected participants were subsequently engaged as faculty in radiation oncology teaching programs across Ukraine. Observers were matched to host institutions based on their clinical specialty, prior training, preferred equipment vendor, and individualized learning objectives.

Before starting their observerships, all participants attended virtual orientation meetings led by organizers and host institutions. These meetings established learning objectives, outlined the structure of the observership, and addressed any logistical questions. Observers typically received comprehensive support, including visa facilitation, sponsorship for travel and accommodation, and a stipend to cover living expenses. These measures ensured equitable access regardless of participants’ financial circumstances.

To evaluate the impact of these observerships, a cross-sectional, survey-based study design was employed. A structured 23-item survey was distributed to all radiation oncology and medical physics observers, with a median distribution time of 9 months post-observership (**Supplementary A**). The survey assessed clinical and procedural knowledge gained, the influence on professional practice, dissemination of knowledge within Ukraine, barriers to implementation, initiation of new projects, and overall satisfaction with the program.

A success score (maximum 10 points) was created as a composite measure of the observer’s post-training impact. It included the implementation of new procedures at the home institution (5 points), initiation of external improvement projects (1 point), informal colleague training (1 point), institutional presentations (1 point), national conference presentations (1 point), and incorporation of knowledge into public educational lectures (1 point). Satisfaction with the observership was also rated on a 10-point scale. Both success and satisfaction scores were analyzed in relation to participant characteristics including time from certification, profession, frequency, and duration of observership.

Descriptive statistics were used to summarize participant characteristics and overall trends. Group comparisons were made using the independent samples non-parametric data Mann-Whitney U test, with p-value < 0.05 as statistically significant.

## Results

A total of 43 Ukrainian medical professionals who completed international radiation oncology observerships responded to the survey, yielding a high response rate of 86.0%. Among them, 28 participants (65.1%) were radiation oncology professionals, including 19 (67.8%) radiation oncologists and 9 (32.1%) medical physicists, representing institutions from across Ukraine. This work presents results exclusively for participants in radiation oncology and medical physics. The median age was 37 years (range: 24–54), and gender distribution was 78.6% female and 21.4% male (**Table 1**). The majority of observership participants were female. This imbalance was partly due to restrictions on international travel for men of military age, who were prohibited from leaving Ukraine during the early phase of the full-scale invasion. While women already represent a substantial portion of the Ukrainian radiation oncology workforce (around 75%) and medical physics workforce (around 40%), these wartime restrictions further limited the participation of male professionals in international training opportunities. The median time from certification was 10 years (range 0 to 22).

**Table 1:** Observers’ characteristics.

Observers characteristics	Number (%)
Age	
Median; range	37; 24 to 54
Gender	
Female	22 (78.6%)
Male	6 (21.4%)
Profession	
Radiation Oncologist	19 (67.9%)
Medical Physicist	9 (32.1%)
Time from certification (years)	
Median; range	10; 0 to 22
Length of observership (weeks)	
Median; range	4; 0.5 to 32
Attendance of professional meeting	
Yes	27 (96.4%)
No	1 (3.6%)
Training institution abroad	
Stanford University Hospital, USA	7 (21.2%)
Miami Cancer Institute/Baptist Health, USA	3 (9.1%)
the Christie Hospital, UK	3 (9.1%)
Northwestern University, USA	3 (9.1%)
Johns Hopkins, USA	2 (6.1%)
Mayo Clinic, USA	2 (6.1%)
Princess Margaret Cancer Center, Canada	2 (6.1%)
University of Pennsylvania, USA	2 (6.1%)
University of Washington Hospital, USA	2 (6.1%)
Icon Group Icon Cancer Centers, Australia	2 (6.1%)
Sunnybrook Health Sciences Centre, Canada	2 (6.1%)
University of California Irvine Hospital, USA	1 (3.0%)
Royal Marsden, UK	1 (3.0%)
Brigham and Women’s Hospital, USA	1 (3.0%)

**Figure 1:**
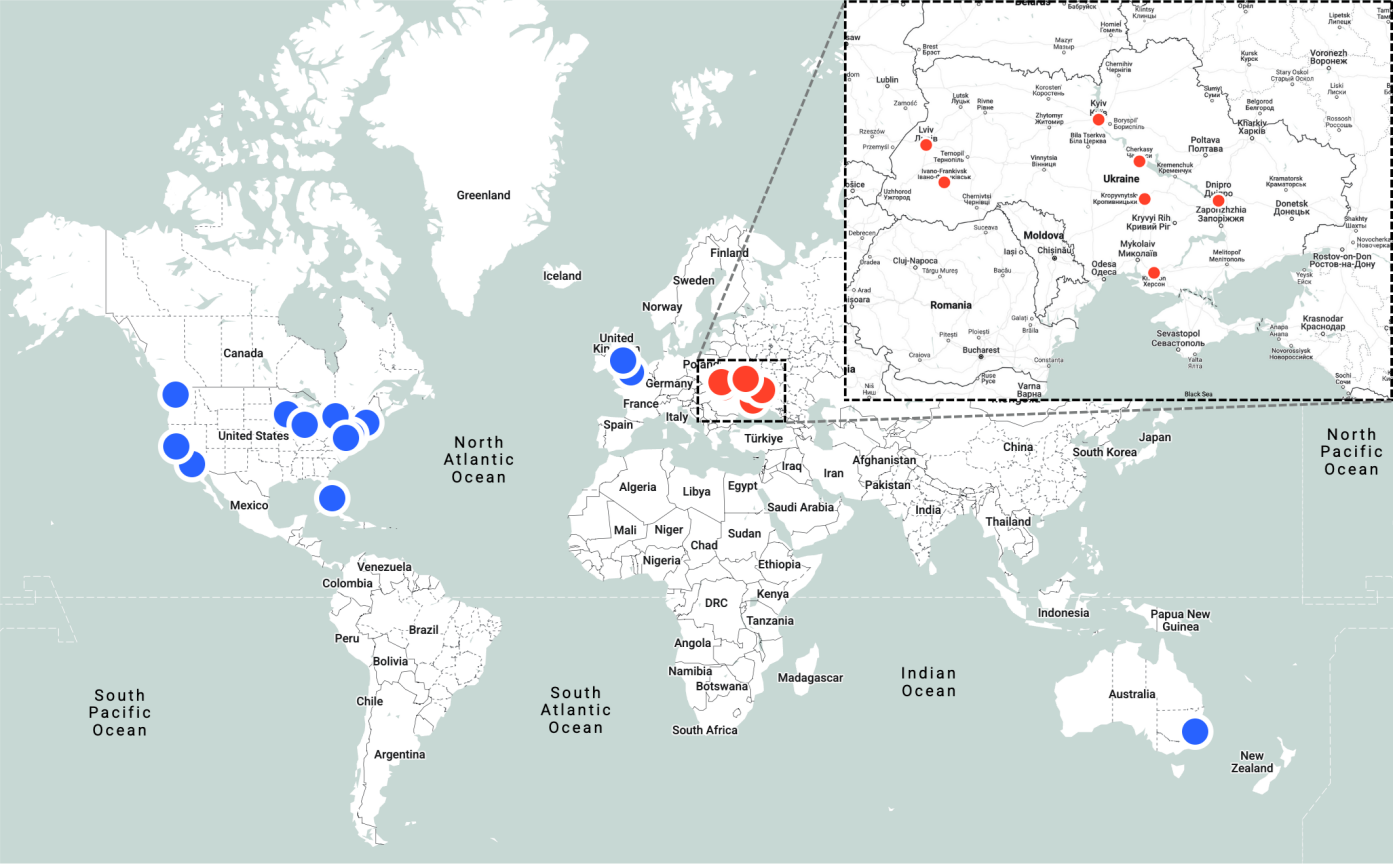
Geographic distribution of observers’ home and training institutions. Blue markers represent the global training institutions across the United States, Canada, the United Kingdom, and Australia where observerships were hosted between 2022 and 2024. The inset highlights the distribution of observers’ home institutions within Ukraine (in red).

The median duration of observerships for radiation oncology professionals was 4 weeks (range: 3 days to 32 weeks). Short-term observerships in our program were intentionally designed to be highly focused and procedure-specific, often concentrating on defined clinical tasks or technologies, whereas longer observerships allowed for broader clinical immersion and longitudinal engagement. Radiation oncology and medical physics participants trained at 14 academic cancer centers in the United States (Stanford University, Northwestern University, Miami Cancer Institute/Baptist Health, Johns Hopkins, Mayo Clinic, University of Pennsylvania, University of Washington Hospital, University of California Irvine Hospital, Brigham and Women’s Hospital), Canada (Princess Margaret Cancer Center, Sunnybrook Health Sciences Centre), Europe (the Christie Hospital, Royal Marsden), and Australia (Icon Group Icon Cancer Centers). **Figure 1** shows the locations of observers’ institutions in Ukraine and abroad. Although observership duration and specific content varied across institutions, all placements were guided by predefined learning objectives aligned with modern radiotherapy standards. This flexibility allowed adaptation to institutional expertise and international practice differences while maintaining core educational goals.

Notably, 96.4% of radiation oncology and medical physics participants also attended a professional conference during their observership. Conferences included ASTRO 2022, AAPM Spring meeting 2023, ASTRO 2023, ABG 2023, Varian Australasian Oncology Summit 2023, ESTRO 2024, CARO 2024, and ASTRO 2024 which enriched their learning experience and provided them an opportunity to see the technology and scientific depth. Participants highlighted the importance of these meetings for exposure to cutting-edge research, team-building, networking, and international collaboration.

**Figure 2:**
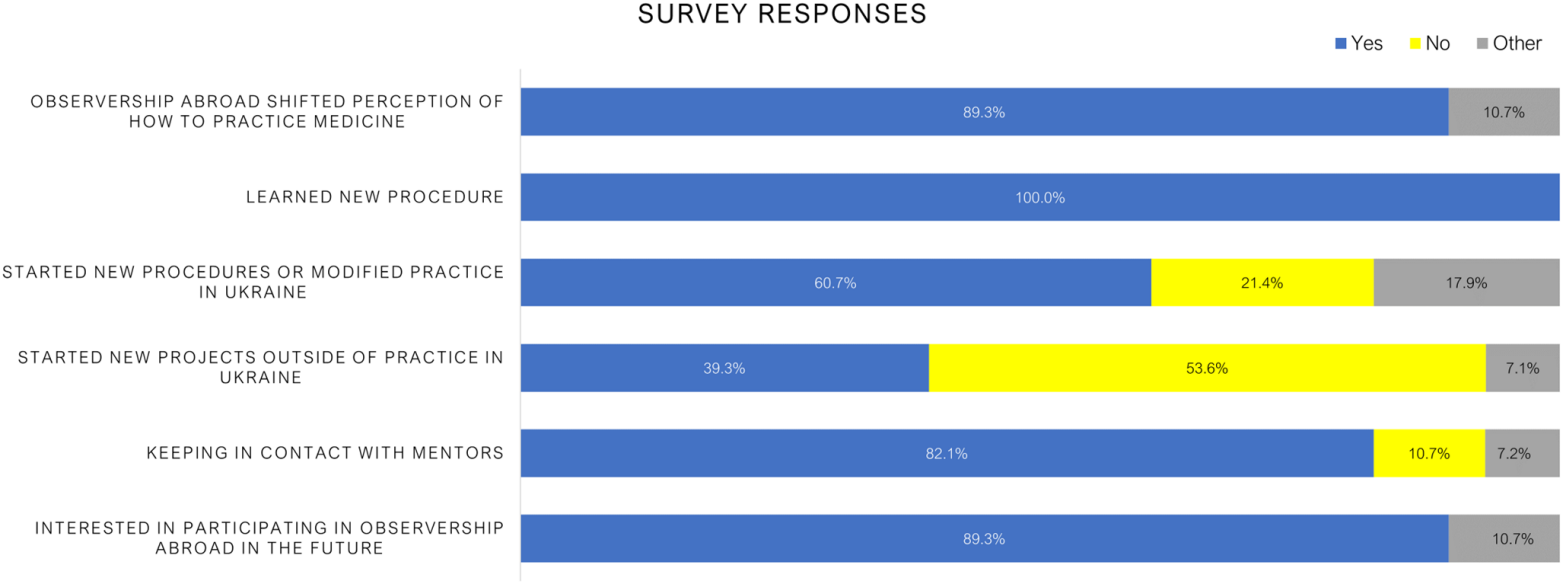
Survey responses highlighting the perceived impact of international observerships among Ukrainian healthcare professionals. The majority of participants reported a shift in their perception of how to practice medicine (89.3%), acquisition of new procedures or techniques (100%), and implementation of new or modified practices upon return to Ukraine (60.7%). Additionally, 82.1% maintained contact with mentors from their host institutions. While knowledge dissemination and institutional change were prevalent, fewer participants (39.3%) reported starting new projects outside of their clinical practice. Interest in future participation remained high, with 89.3% expressing willingness to take part in additional observerships.

The observerships were widely viewed as transformative: 100% of radiation oncology professionals reported learning new procedures or technologies, while 89.3% noted a shift in their perception of how to practice radiotherapy. The mean satisfaction score for the observership experience was 9.5 out of 10 (median 10, IQR 9 -10), reflecting the strong perceived value and impact of the program as shown in **Figure 2**. Details on “Other” answers are provided in **Supplementary B**.

**Figure 3:**
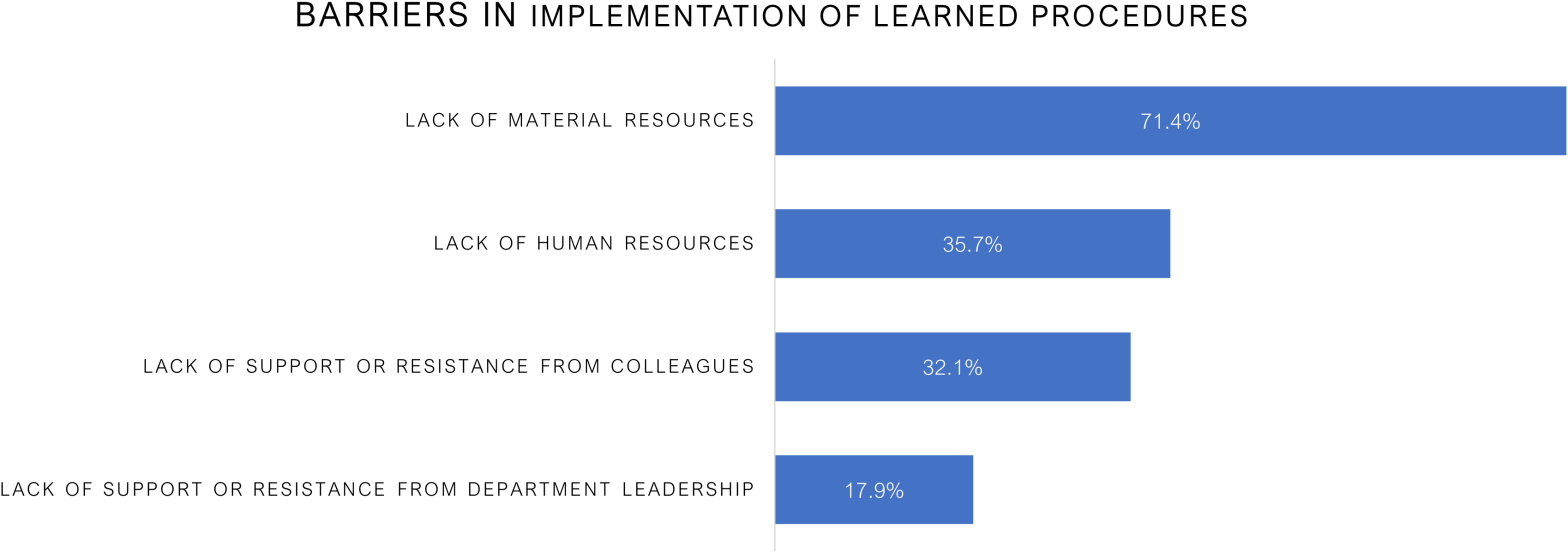
Reported barriers to implementation of learned procedures among radiation oncology observers. The most common challenge was lack of material resources (71.4%), followed by insufficient human resources (35.7%), lack of support or resistance from colleagues (32.1%), and limited support from department leadership (17.9%). These findings underscore the systemic constraints faced by Ukrainian professionals in translating international training into clinical practice.

Upon return to Ukraine, 60.7% successfully implemented new procedures in their clinical practice. These included transitioning from Co-60 to linear accelerators, introducing hypofractionation, initiating SBRT programs, improving contouring, dosimetry protocols, quality assurance processes, and launching radiotherapy plan review procedures. The most commonly reported obstacles in new procedure implementation included lack of material resources (71.4%), such as absence of linear accelerators or advanced planning systems, followed by insufficient human resources (35.7%), and limited support from colleagues (32.1%) or department leadership (17.9%) (**Figure 3**).

Beyond direct improvements in patient care, 39.3% of radiation oncology professionals launched new initiatives outside their routine clinical duties, applying insights gained from their observerships to broader educational, research, or advocacy efforts.

**Figure 4:**
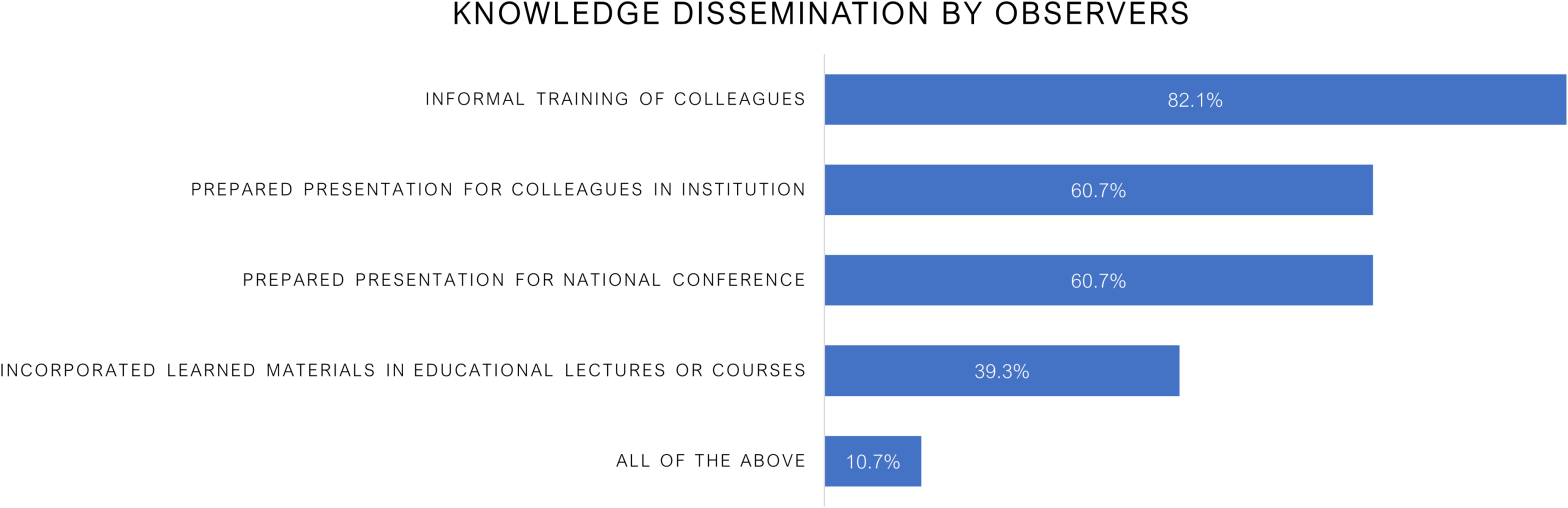
Knowledge dissemination activities among radiation oncology observers following international observerships. The majority of participants engaged in informal training of colleagues (82.1%) and delivered presentations at their institutions and national conferences (60.7%). Additionally, 39.3% incorporated materials into educational lectures or courses, and 10.7% participated in all dissemination activities, reflecting strong commitment to sharing acquired knowledge within Ukraine’s oncology community.

Knowledge dissemination was high: 82.1% provided informal training to colleagues, 60.7% delivered presentations at their home institutions or national conferences, and 39.3% integrated learned materials into educational lectures or courses (**Figure 4**). Notably, 10.7% engaged in all of these dissemination activities, further amplifying the reach of their training.

Additionally, 82.1% maintained active mentorship contact with their host institutions, fostering continued professional development and international collaboration.

Overall average satisfaction was 9.5 (median 10.0, IQR 9.0–10.0) and was uniformly high across subgroups (**Table 2**) with no significant differences observed by profession (p = 0.11), length of observership (p = 0.48), or frequency of participation (p = 0.85). Medical physicists reported slightly higher satisfaction (9.9, median 10.0, IQR 10.0–10.0) compared to radiation oncologists (9.3, median 10.0, IQR 9.0–10.0), although this difference was not statistically significant. Success scores demonstrated greater variability, with an overall mean of 6.1 (median 7.0, IQR 5.0–8.0). Observers with <10 years since certification reported significantly lower success scores (mean 4.7, median 5.5, IQR 2.0–7.0) compared to those with ≥10 years (mean 7.4, median 8.0, IQR 6.0–9.0; p = 0.01). No significant differences in success scores were found by profession (p = 0.18), length of observership (p = 0.45), or frequency of participation (p = 0.53).

**Table 2:** Observers’ average satisfaction and success scores out of 10 relative to observers’ characteristics.

Observers characteristics	Satisfaction score out of 10	p-value	Success score out of 10	P-value
Time from certification (years)		0.39		0.01*
< 10 years	9.3 (median 10.0, IQR 8.8-10.0)		4.7 (median 5.5, IQR 2.0-7.0)	
> = 10 years	9.6 (median 10.0, IQR 9.3-10.0)		7.4 (median 8.0, IQR 6.0-9.0)	
Profession		0.11		0.18
Radiation Oncologist	9.3 (median 10.0, IQR 9.0-10.0)		6.7 (median 7.0, IQR 5.8-9.0)	
Medical Physicist	9.9 (median 10.0, IQR 10.0-10.0)		5.0 (median 5.0, IQR 2.0-7.0)	
Length of observership (weeks)		0.48		0.45
< 4 weeks	9.4 (median 10.0, IQR 9.0–10.0)		5.7 (median 7.0, IQR 3.0–7.5)	
> = 4 weeks	9.6 (median 10.0, IQR 9.5–10.0)		6.7 (median 7.5, IQR 5.0–8.8)	
Frequency of observership		0.85		0.53
Once	9.5 (median 10.0, IQR 9.0–10.0)		5.8 (median 7.0, IQR 3.5–8.0)	
Twice or more	9.5 (median 10.0, IQR 9.0–10.0)		6.7 (median 7.0, IQR 5.0–9.0)	
Total				
	9.5 (median 10.0, IQR 9.0-10.0)		6.1 (median 7.0, IQR 5.0-8.0)	

* denotes statistical significance p<0.05.

## Discussion

To our knowledge, this study represents the largest effort to evaluate the impact of international observerships on radiation oncology professionals from LMIC/UMIC during wartime. It highlights the transformative influence of global collaboration on clinical practice, education, and institutional development within radiotherapy. Such initiatives may represent a critical strategy for sustaining and expanding healthcare capacity in settings affected by war and other conflict-related disruptions.

### Comparison with prior literature in radiation oncology

The existing literature on international observerships in radiation oncology is notably scarce. The European School of Oncology’s (ESO) Clinical Training Centers (CTC) fellowship program offers a relevant parallel, having delivered 74 fellowships across oncology disciplines over seven years, including 11 in radiation oncology (15). Fellows reported very high satisfaction (95.5% rating the program as “excellent” or “very good”) and universal achievement of learning objectives, reflecting experiences consistent with our study. Importantly, the ESO model, like ours, emphasized mentorship, integration into multidisciplinary care, and exposure to advanced technologies in leading centers.

The experience of establishing the first structured, board-certified radiation oncology training program in Iraq provides an important parallel to our findings (16). Despite prolonged conflict and limited local expertise, Iraq successfully leveraged international partnerships, visiting experts, and formal agreements with global institutions to supplement national training capacity. These efforts resulted not only in sustained specialist education but also in the launch of multidisciplinary oncology courses and expansion of local expertise across radiation oncology, medical physics, and clinical oncology. Similar to our observations, this model demonstrates how targeted international engagement through short-term expert visits, institutional networking, and structured educational initiatives can meaningfully strengthen radiotherapy training programs in conflict-affected and resource-constrained settings.

A scoping review by Karim et al. (11) documented only 25 full-text oncology training initiatives in LMICs published over seven decades, with just two specifically focused on radiation oncology: Agrawal et al. (17), who piloted a self-guided contouring module with feedback to support transition from 2D to 3D planning, and Abugideiri et al. (18), who evaluated the role of telemedicine for radiation oncology training in a developing country. These limited examples underscore how underrepresented radiotherapy education remains despite its central role in cancer treatment.

**Figure 5:**
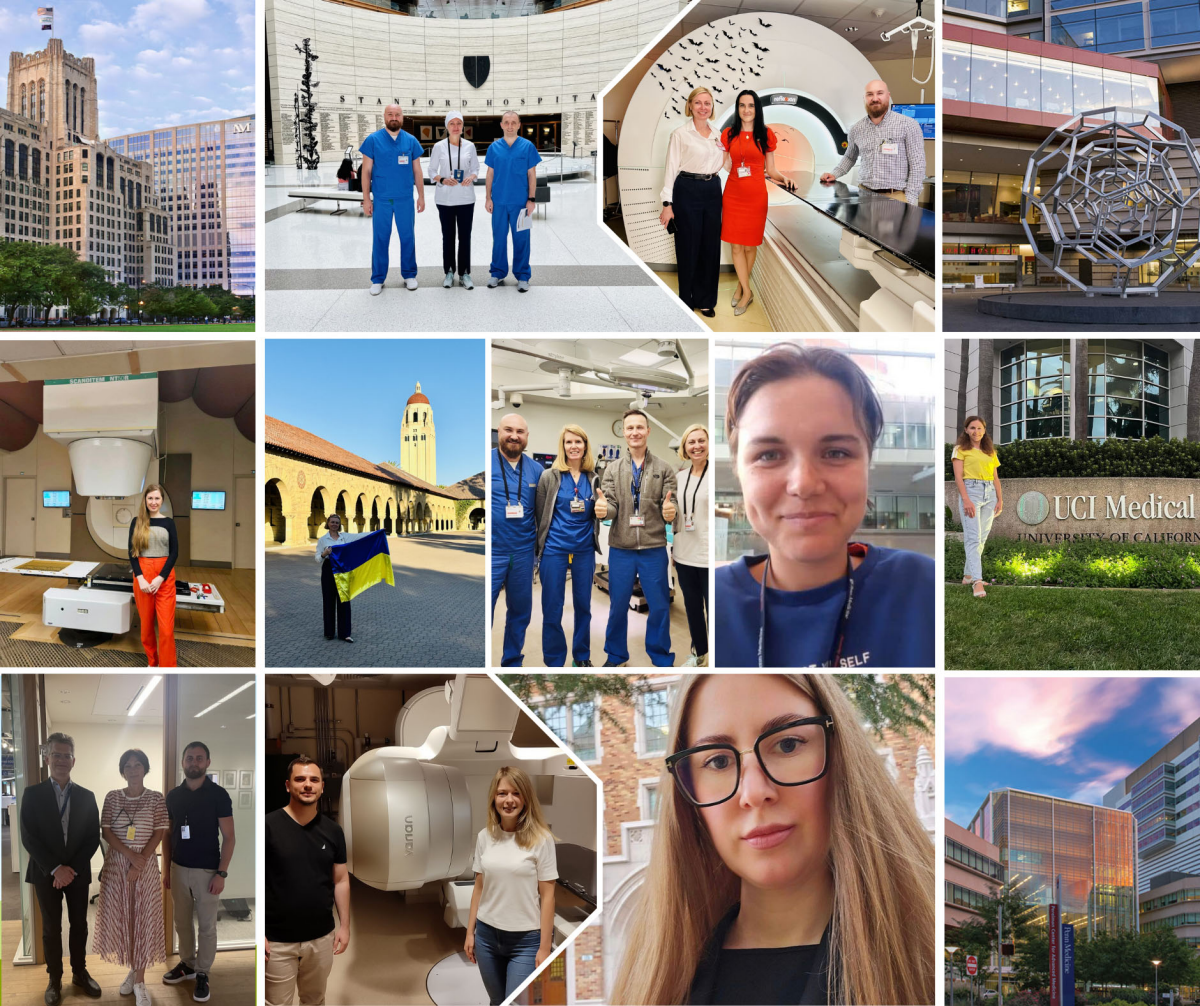
Ukrainian radiation oncologists and medical physicists participating in international observerships at leading cancer centers abroad, gaining hands-on experience, mentorship, and exposure to advanced radiotherapy techniques with the goal of strengthening oncology care in Ukraine.

**Figure 6:**
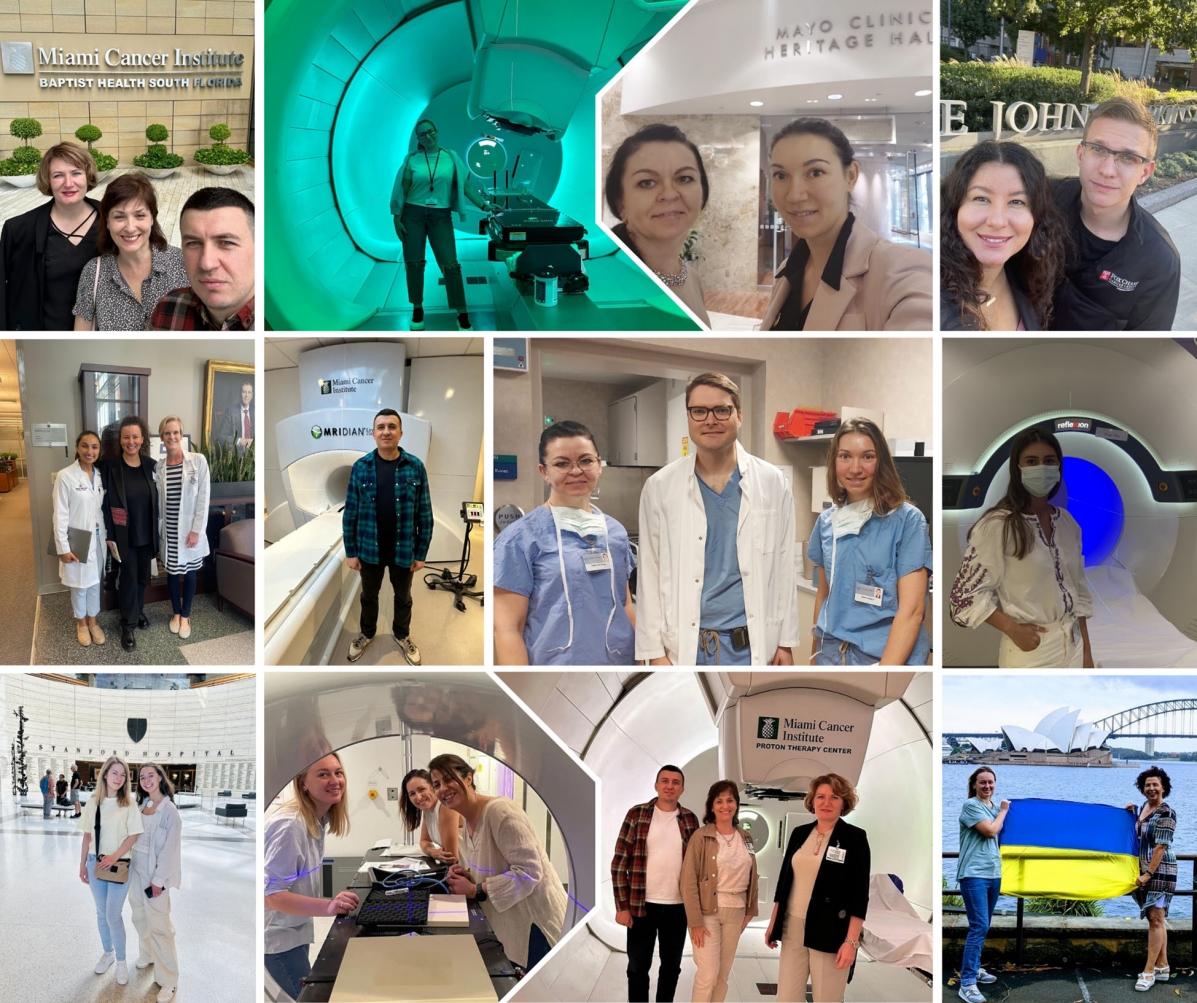
Ukrainian radiation oncologists and medical physicists participating in international observerships at leading cancer centers abroad, gaining hands-on experience, mentorship, and exposure to advanced radiotherapy techniques with the goal of strengthening oncology care in Ukraine.

As summarized by Karim et al. (11), most LMIC-focused initiatives have historically prioritized physicians and short-term skill acquisition. By contrast, the Help Ukraine Group program expanded its scope to include both physicians and medical physicists (**Figures 5**, **6**), with future plans for radiation therapist training. This broader inclusion not only ensured high satisfaction and procedural learning but also fostered a transformative shift in clinical mindset supporting multidisciplinary, patient-centered, and evidence-based approaches to cancer care, as previously described in global training reports (19).

### Domains of impact

Our structured assessment of observership participants identified three major domains of impact: clinical knowledge and skill acquisition, implementation of new clinical practices, and dissemination of knowledge through educational activities in Ukraine.

### Clinical knowledge and skill acquisition

In our study, participants acquired significant exposure to advanced technologies such as hypofractionated intensity modulated radiotherapy (IMRT), volumetric modulated arc therapy (VMAT), stereotactic body radiotherapy (SBRT), adaptive radiotherapy (ART) and 3D and magnetic resonance imaging (MRI)-guided/ultrasound (US)-guided high dose rate (HDR) brachytherapy for various cancers, techniques largely unavailable or underutilized in Ukraine. Many were exposed to the cutting-edge equipment such as MRI-linac, on-line adaptive CT-linac, positron emission tomography (PET)-linac, proton therapy machines, and were introduced to novel dosimetric procedures and workflows using AI-contouring, planning, 3D printing, enhanced quality and safety procedures, etc. This knowledge shifted not only technical capabilities but also the observers’ mindset, fostering a more multidisciplinary, patient-centered, evidence-based, and cutting-edge technology approach to care. Respondents reported increased confidence in decision-making and emphasized the importance of structured care pathways and modern radiotherapy standards. This outcome is challenging to achieve with virtual training alone, underscoring the unique value of immersive, in-person observerships.

### Implementation of new practices

There were numerous barriers reported in implementing new procedures including lack of equipment (71.4%), staff shortages (35.7%), and insufficient institutional support (32.1% from colleagues, 17.9% from leadership). These outcomes echo global oncology literature, which emphasizes that translation of knowledge into practice in LMICs is frequently constrained by limited infrastructure and institutional support (11).

Despite facing systemic limitations, 60.7% of participants successfully implemented new procedures or modified existing practices. While the rapid modernization of Ukraine’s radiotherapy infrastructure such as the installation of 24 linear accelerators and procurement of more offers promise, it also magnifies the need for widespread training. Observers noted critical knowledge gaps and a lack of confidence among colleagues during the shift to IMRT and other modern techniques, underscoring the need for scaling educational efforts. Help Ukraine Group has since organized, translated or facilitated 15 virtual courses and 25 on-site training courses in Ukraine to fill the need to some extent.

### Knowledge dissemination

Participants were encouraged from the outset to share their learnings with colleagues upon return to Ukraine, both informally within their departments and formally through presentations, lectures, and training sessions. Our findings demonstrate that these expectations were largely fulfilled as the knowledge dissemination was widespread: 82.1% provided informal training, 60.7% gave institutional/national presentations, 39.3% incorporated materials into lectures, and 10.7% engaged in all activities. Approximately 40% of respondents reported initiating projects outside of their home institution, including co-developing a brachytherapy course for RTTs with the European Brachytherapy Academy, serving as moderators or instructors in various Help Ukraine Group activities as instructors and moderators in the HUG/Oncohub course on Modern Radiotherapy and Case Review with ASTRO Experts course, conducting practical training workshops in Ukraine for radiation oncologists, medical physicists, and radiation therapists, translation of educational courses and books, and collaborating with IAEA initiatives. Several observers collaborated on research projects and participated in writing manuscripts (20–22). Collectively, these activities amplified the observership impact beyond individual participants to broader institutional and national capacity.

### Determinants of satisfaction and success

Overall satisfaction was uniformly high (mean 9.5/10, median 10, IQR 9–10). However, success in implementing practices varied with experience. Professionals ≥10 years post-certification reported significantly higher success (mean 7.4 vs. 4.7, p = 0.01). This suggests that senior clinicians may be better positioned to overcome systemic barriers and influence institutional change. Conversely, early-career professionals, though enthusiastic, may require additional mentorship and structural support to translate knowledge into practice. Importantly, no differences were observed by profession, observership length, or frequency, suggesting broad applicability of the model. These findings argue for tailoring post-observership support to career stage, ensuring younger professionals are empowered to enact sustainable change.

### Mentorship and longitudinal support

A defining strength of our program was sustained mentorship: 82.1% maintained contact with host institutions. Building on this, a structured remote mentoring program was initiated in 2024 to support two Ukrainian centers, Precarpathian Clinical Oncology Center and Clinical Center for Oncology Cherkasy, as they transitioned from cobalt-based radiotherapy to linear accelerators. This initiative, developed in collaboration with HUG, the Australasian College of Physical Scientists and Engineers in Medicine (ACPSEM), Australian Society of Medical Imaging and Radiation Therapy (ASMIRT) and Elekta, and led by Dr. Natalka Suchowerska complemented the observership model by providing sustained, context-specific guidance through weekly tutorials, treatment plan reviews, and interactive discussions. The program effectively strengthened both clinical and physics competencies, particularly in contouring, immobilization, simulation protocols, treatment planning, and radiation dosimetry.

The Australian-led program began with weekly virtual tutorials and practical assignments, later expanding to two to three weekly sessions including panel discussions, treatment plan reviews, and collaborative problem-solving. Surveys from participants (12 radiation oncologists and 5 medical physicists) confirmed high professional value, relevance, and strong participant engagement, with the small-group format mitigating language barriers. Radiation oncologists reported greater confidence in contouring (OAR margins, target volume delineation, positioning, CT simulation, and pre-treatment preparation), while medical physicists highlighted improvements in treatment planning (3D/IMRT), water phantom use, small-field dosimetry, and LINAC-based radiotherapy.

Despite its time-intensive nature, the initiative fostered high engagement, mitigated language barriers through a small-group format, and offered immediate clinical relevance within the constraints of wartime practice. Plans for expansion of Australian-led mentorship program to additional centers underscore its perceived value and sustainability.

Our findings parallel those from programs such as Project ECHO and other tele-mentoring efforts, which stress that sustained professional relationships are key to bridging initial training into long-term capacity (23). Our structured remote mentoring program, supporting Ukrainian centers during cobalt-to-linac transitions, reflects similar approaches seen in Uganda and East Africa, where hybrid in-person and remote formats proved particularly valuable in resource-constrained contexts (24).

### Recommendations for program improvement

Participants suggested that future observerships incorporate more hands-on training, particularly in prostate brachytherapy, adaptive therapy, and contouring. Since hospital policies often restrict observers from direct involvement in patient care, it is essential to provide structured opportunities for practice using phantoms and mock cases to reinforce skills and enhance learning. Matching trainees with mentors, enhancing collaboration with Ukrainian institutions, and facilitating trainee-team formats were other common feedback themes. Expanded opportunities for clinical research, increased exposure to multidisciplinary tumor boards, and training in protocol development were also recommended. Expanding exposure to tumor boards, research methods, and protocol development also resonates with findings from oncology fellowships in Rwanda and Bangladesh, which demonstrated the added value of integrating clinical training with research and leadership development (25, 26). Similarly, the St. Jude International Outreach Program underscores that pairing clinical training with research, policy engagement, and institutional development ensures sustainability beyond individual capacity-building (27). Building on this model, the Help Ukraine Group and ASTRO International Committee launched a Research Methodology Course with mentorship for Ukraine’s radiation oncology and medical physics community, resulting in 16 proposed and 5 funded research projects to date. Inspired by the outcomes on the observership project, Help Ukraine Group is now organizing 42 additional team-based observerships for radiation oncologists, medical physicists, and radiation therapy technologists (RTTs) across the European institutions in 2025-2026. This multidisciplinary model better reflects clinical practice and supports comprehensive capacity building. If successful, the total number of observers will represent approximately 10% of the entire Ukrainian radiation oncology workforce. This initiative may approach a critical mass where individual capacity-building begins to catalyze broader systemic change within the radiation oncology community. This raises the important question of whether such a threshold together with the arrival of modern equipment could serve as a tipping point for the renaissance of radiation therapy transformation in Ukraine.

### Limitations

This study has several limitations. First, selection bias may have occurred as participants were highly motivated professionals, potentially overestimating the program’s impact. Second, self-reported data is subject to recall bias as the survey was distributed at a median nine months post observerships and social desirability bias. The short and inconsistent follow-up period limits the ability to assess the long-term sustainability of the implemented changes. To address this limitation, we have implemented a longitudinal evaluation framework that includes brief surveys during the observership, immediately after completion, and at nine months post observership, with plans for longer-term follow-up. Third, the lack of a control group and variability in follow-up periods limit attribution of outcomes solely to the observerships. Lastly, while implementation was reported, long-term clinical outcomes were not measured.

## Conclusion

International observerships were instrumental in advancing the skills and expertise of Ukrainian cancer care professionals amid the ongoing war. Despite the challenges of conflict, participants achieved meaningful progress in clinical practice, medical education, and the adoption of new procedures. This initiative underscored the resilience and determination of Ukraine’s medical community, emphasizing the critical value of sustained professional development during crises. The success of these observerships offers a scalable model for similar efforts in other low- and middle-income and upper-middle-income countries.

## Data Availability

The raw data supporting the conclusions of this article will be made available by the authors, without undue reservation.
