# Supplementary material for: Advancing radiation oncology care in Ukraine during the war: impact of international observerships on professional development and clinical practice
Source: Front Oncol. 2026 Mar 25;16:1752691. doi: 10.3389/fonc.2026.1752691 (PMC13056684; doi:10.3389/fonc.2026.1752691)
Supplement: Supplementary file 1 [file DataSheet1.docx]

Evaluation of Training Abroad Programs for Ukrainian Medical Professionals

Start of Block: Default Question Block

Q1 Last Name, First Name

________________________________________________________________

Q2 Your age at the time of observership

________________________________________________________________

Q3 Your gender

________________________________________________________________

Q4 Your institution in Ukraine

________________________________________________________________

Q5 Your specialty

- Anesthesiologist
- Bone Marrow Transplant oncologist
- Intensivist
- Internal medicine
- Medical Oncologist
- Medical Physicist
- Neurology/Neurosurgery
- Pediatric medical oncologist
- Psychiatry
- Psychology
- Radiation Oncologist
- Radiologist
- Rehabilitation Medicine
- Surgeon - General
- Surgeon - Otolaryngology
- Surgeon - Oral and Maxiofacial
- Surgeon - Thoracic and abdominal
- Surgeon - Plastic
- Surgeon - Burn
- Surgeon - Orthopedic
- Surgeon - Trauma
- Surgeon - Transplant
- Surgeon - Other __________________________________________________
- Other __________________________________________________

Q6 Years after specialty certification

________________________________________________________________

| Page Break |  |
| --- | --- |

Q7 Institution you trained abroad. If you trained at multiple institutions, please fill out the separate survey per each institution.

- Boston Medical Center
- Brigham and Women's Hospital
- Brown University Hospital
- Fox Chase Hospital
- ICON Group affiliated Hospital
- Johns Hopkins
- Massachusetts General Hospital
- Mayo Clinic
- MD Anderson Cancer Center
- Medical College of Wisconsin
- Miami Cancer Institute/Baptist Health
- Northwestern Memorial Hospital
- Oklahoma University
- Princess Margaret Hospital
- Stanford University Hospital
- University of California Irvine Hospital
- University of Oklahoma
- University of Pennsylvania
- University of Washington Hospital
- University of Wisconsin
- USA Uniformed Services University
- Vanderbilt University Medical Center
- Yale University
- Other __________________________________________________

Q8 Country you trained in?

- USA
- Canada
- Australia
- UK
- Other __________________________________________________

Q9 Length of training (in weeks)

________________________________________________________________

Q10 Dates of training

________________________________________________________________

Q11 Did you attend the national/international conference before, after, or during your training? If yes, specify the name of the conference

- Yes __________________________________________________
- No
- Other __________________________________________________

| Page Break |  |
| --- | --- |

Q12 Evaluate the overall effectiveness of training (1 - least effective, 10 - most effective)

|  | 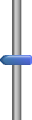 | 0 (0)  1 (1)  2 (2)  3 (3)  4 (4)  5 (5)  6 (6)  7 (7)  8 (8)  9 (9)  10 (10) |
| --- | --- | --- |

Q13 Did the observership abroad shifted your perception of how to practice medicine? If yes, what specifically changed it the most?

- Yes __________________________________________________
- No
- Other __________________________________________________

Q14 Did you learn any new procedures during your observership abroad? If yes, what exactly?

- Yes __________________________________________________
- No
- Other __________________________________________________

Q15 How did the training abroad change your practice in Ukraine?

________________________________________________________________

________________________________________________________________

________________________________________________________________

________________________________________________________________

________________________________________________________________

Q16 How are you transferring knowledge you acquired abroad to your peers in Ukraine?

- Prepared presentation for colleagues in institution
- Prepared presentation for national conference
- Incorporated learned materials in educational lectures I am giving
- Organized a training course
- Informal training of colleagues
- All of the above
- Other __________________________________________________

Q17 Have you started new procedures or modified your practice in Ukraine based on what you learned during your training abroad? If yes, please specify which procedures.

- Yes __________________________________________________
- No
- Other __________________________________________________

Q18 Did you experience any difficulty with implementing the knowledge you acquired abroad to your practice in Ukraine? Why?

- Yes, lack of support or resistance from department leadership
- Yes, lack of support or resistance from colleagues
- Yes, lack of material resources (e.g., equipment, software)
- Yes, lack of human resources (e.g., insufficient staffing)
- Yes, other reasons __________________________________________________
- No

Q19 Have you started new projects outside of your practice in Ukraine based on what you learned during your training abroad? If yes, please elaborate.

- Yes __________________________________________________
- No
- Other __________________________________________________

Q20 Do you keep in contact with your training program mentors? If yes, please specify how.

- Yes __________________________________________________
- No
- Other__________________________________________________

Q21 Did your hospital in Ukraine provide financial support for the observership abroad?

- Yes __________________________________________________
- No
- Other __________________________________________________

Q22 Please describe the areas of improvement for the training abroad program.

________________________________________________________________

________________________________________________________________

________________________________________________________________

________________________________________________________________

________________________________________________________________

Q23 Are you interested in participating in observership abroad in the future?

- Yes
- No
- Other __________________________________________________
