# Supplementary material for: Advancing radiation oncology care in Ukraine during the war: impact of international observerships on professional development and clinical practice
Source: Front Oncol. 2026 Mar 25;16:1752691. doi: 10.3389/fonc.2026.1752691 (PMC13056684; doi:10.3389/fonc.2026.1752691)
Supplement: Supplementary file 2 [file DataSheet2.docx]

Details on “Other” responses

1. **Did the observership abroad shifted your perception of how to practice medicine? If yes, what exactly impressed you the most? Other - Text**

- Provision of equipment and auxiliary means for radiotherapy
- Given that before visiting the clinic and after returning, we work in the department on cobalt machines, it is difficult to apply the seen treatment techniques in practice. The impact caused by this internship will be realized in the near future, when the first patients will come to be treated at the linear accelerator. The experience of how doctors conduct patient consultations and organize work schedules is very useful.

1. **Have you started new procedures or modified your practice in Ukraine based on what you learned during your training abroad? If yes, please specify which procedures.** **Other - Text**

- **I am in the process of becoming a radiation oncologist.**
- **My colleagues and I have improved our treatment planning skills, particularly in the use of hypofractionation.**
- **We have started using hypofractionation more frequently.**
- **At the moment, no; however, this will become possible once work on the linear accelerator begins.**
- **This is an ongoing process, and I continue to reflect on and apply what I observed during my clinical work.**

1. **Have you started new projects outside of your practice in Ukraine based on what you learned during your training abroad? If yes, please elaborate. Other – Text**

- **Not yet, but I am thinking about how to best use the knowledge I gained.**
- **Currently no, but I am confident that I will have this opportunity in the future.**

1. **Do you keep in contact with your training program mentors? If yes, please specify how. - Other – Text**

- **I attend lectures, participate in sponsored courses, and maintain contact with colleagues for advice and guidance.**
- **I have established some professional contacts but have not yet used them.**

1. **Are you interested in participating in observership abroad in the future? - Other – Text**

- **I do not have an answer at this time.**
- **Possibly.**
